# Clonal hematopoiesis detection by simultaneous assessment of peripheral blood mononuclear cells, blood plasma, and saliva

**DOI:** 10.1172/JCI191256

**Published:** 2025-06-19

**Authors:** Caitlin M. Stewart, Sonya Parpart-Li, James R. White, Mitesh Patel, Oliver Artz, Michael B. Foote, Erika Gedvilaite, Michelle F. Lamendola-Essel, Drew Gerber, Rohini Bhattacharya, Justin M. Haseltine, Kety Huberman, Kelly L. Bolton, Ross L. Levine, Luis A. Diaz

**Affiliations:** 1 Memorial Sloan Kettering Cancer Center, New York, New York, USA.; 2 GRAIL, Menlo Park, California, USA.; 3 Resphera Biosciences, Baltimore, Maryland, USA.; 4 Grossman School of Medicine, New York University, New York, New York, USA.; 5 Emory University, Atlanta, Georgia, USA.; 6 Eurofins, Fremont, California, USA.; 7 Washington University, St. Louis, Missouri, USA.

**Keywords:** Genetics, Hematology, Oncology, Bioinformatics, Molecular diagnosis, Molecular genetics

**To the Editor:** Most genomic analyses of biofluids rely on DNA from a single source, like peripheral blood, which delivers reproducible results when reporting genomic alterations highly represented in the specimen. However, there may be value from simultaneously measuring somatic mutations in multiple biofluids from the same individual. We posited that observation of the same mutation across biofluids could increase confidence in positive mutation calls, improve the limit of detection, and perhaps reveal important clinical relationships.

To test this, we used clonal hematopoiesis (CH), a mutational profile commonly found in hematopoietic cells. CH results from the expansion of hematopoietic stem/progenitor cells and their differentiated progeny, which harbor ≥1 somatic mutation and is associated with advanced age (1). Many of these mutations are identical to those in acute myeloid leukemia (AML) and are associated with increased risk of cardiovascular disease, as well as AML itself (1), and can be tracked in PBMC DNA or in cell-free DNA (cfDNA) from plasma.

We derived DNA from PBMCs (buffy coat) and saliva (2) and cfDNA from plasma, designed a high-coverage capture panel to detect CH mutations, and performed an analytical validation study of the assay targeting the coding regions of 19 genes associated with CH (1). Our assay identified 94.71% of variants at standard depth (1,000X) and 100% of cases at high depth (10,000X) and was highly concordant with matched buffy coat sequencing data using a targeted tissue panel (MSK-IMPACT) (3, 4) (Supplemental Figure 1, A and B, and Supplemental Table 1; supplemental material available online with this article; https://doi.org/10.1172/JCI191256DS1). The limits of detection were 1% variant allele frequency (VAF) for standard depth and 0.3% VAF for high depth (Supplemental Figure 1, C and D, and Supplemental Table 2).

Results of analysis of 60 individuals with CH showed high concordance between mutations detected in the buffy coat and saliva (*R*^2^ = 0.95) and a similar level of concordance (*R*^2^ = 0.86) found in mutations detected in cfDNA and the buffy coat (Figure 1A). There were no meaningful differences in VAFs between the 3 sources: buffy coat (mean VAF, 8.16%), cfDNA (mean VAF, 7.78%), and saliva (mean VAF, 7.00%, Figure 1B).

Given the relationship of CH to AML, we sequenced the buffy coat and cfDNA from 5 patients with myelodysplastic syndromes (MDS) and 6 patients with AML for CH variants (Figure 1, C and D). In both AML and MDS, there was high concordance in the mutations detected between the buffy coat and cfDNA. VAFs between buffy coat and cfDNA in AML were also concordant. However, in the cases of MDS, the VAFs in cfDNA were significantly (*P* = 0.0082) higher than in the buffy coat, which was not explained by an increase in peripheral circulating blasts (Supplemental Table 3). In AML, malignant blasts were present in the circulation of our patients, whereas in MDS, they were confined to the bone marrow and absent in the circulation, which may explain the relatively lower VAF in the buffy coat in MDS.

We next evaluated whether combining sequencing data from multiple biofluid types would increase confidence of variant calls. We found higher confidence scores and higher sensitivity for calling mutations at low VAFs when assessing the combination of 2 and 3 biofluids (Figure 1E), albeit at a slightly lower specificity (Supplemental Figure 1I).

Using CH as a model, simultaneous assessment of somatic mutations from multiple biofluids identified practical clinical and technical applications that are likely applicable to other biologic and clinical scenarios where mutations are measured in biofluids. Integration of the differences in mutations in DNA, and potentially other biomolecules, from different biofluid sources will likely demonstrate differences that are measurable and actionable. In the case of hematologic malignancies, differences in VAF and diversity in circulating myeloblasts when compared with cfDNA provide insights into disease progression and can help distinguish malignant from benign clonal events in the blood, in essence providing the ratio between mutant cfDNA (plasma DNA) and circulating tumor cells (buffy coat). Whether simultaneous assessment of somatic mutations from multiple biofluids is applicable to other biologic and clinical scenarios remains to be seen.

## Supplementary Material

Supplemental data

Supporting data values

## Figures and Tables

**Figure 1:**
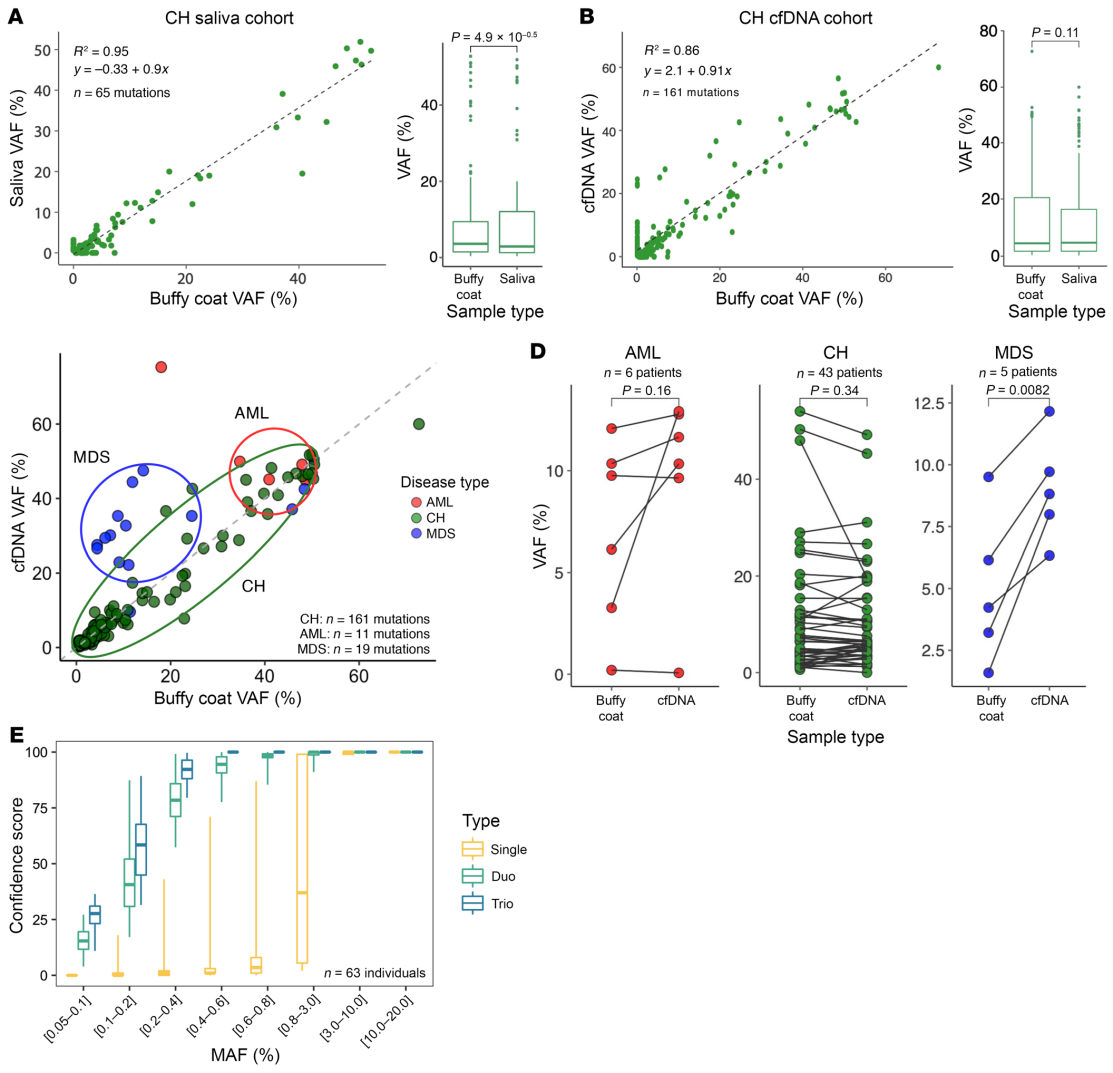
Concordance of mutations in CH, MDS, and AML between biofluids and sensitivity/specificity of multiple biofluid calls. (**A**) Scatter plot and box plot of VAFs in buffy coat versus saliva for CH, showing that saliva has lower VAFs than buffy coat (*P* = 4.9 × 10^–5^, 95% CI [–4.21, 1.90]). (**B**) Scatter plot and box plot of VAFs in buffy coat versus cfDNA in CH, showing nonsignificant differences in buffy coat and cfDNA VAFs (*P* = 0.11, 95% CI [–2.99, 3.13]). (**C**) Scatter plots of each AML and MDS sample, comparing the buffy coat and cfDNA VAFs. (**D**) Dot plots of the VAFs in buffy coat versus cfDNA in AML, CH, and MDS. (**E**) Observed sensitivity when incorporating 1 (single; yellow), 2 (duo; green), or 3 (trio; blue) biospecimens to establish a mutation call. (**A**–**C**) The gray line represents *x* = *y*. Comparisons were done using paired 2-tailed *t* tests. Boxes indicate interquartile range, middle bars denote the median, whiskers represent minimum and maximum values, and dots indicate outliers.

## Version 1 06/19/2025

In-Press Preview

## Version 2 08/15/2025

Electronic publication
