# Supplementary material for: Clonal hematopoiesis detection by simultaneous assessment of peripheral blood mononuclear cells, blood plasma, and saliva
Source: J Clin Invest. 2025 Jun 19;135(16):e191256. doi: 10.1172/JCI191256 (PMC12352887; doi:10.1172/JCI191256)
Supplement: Supplemental data [file jci-135-191256-s128.pdf]

Clonal hematopoiesis detection by simultaneous assessment of peripheral blood mononuclear cells, blood plasma, and saliva

Supplementary Material

Materials and Methods

Sex as a biological variable

Sex was not considered as a biological variable, both sexes of patients and healthy controls were used.

Study Approval

Patients were consented under IRB 12-245 or 06-107 and analysis was done under IRB 18-288 and 21-036 at Memorial Sloan Kettering Cancer Center (MSKCC).

Sample collection and processing

Whole blood and blood plasma from healthy individuals who were younger than 40 years old (to decrease the likelihood of the presence of CH variants) were purchased from BioIVT. Blood and saliva were collected from CH, AML and MDS patients at MSKCC. MSK-IMPACT was performed on whole blood DNA to identify CH patients for inclusion in the study (1). Individuals with CH have received previous cancer treatment at MSKCC. Saliva was collected from MSKCC patients in Oragene OGR-500 tubes (DNA Genotek) and stored at room temperature per manufacturer guidelines. Whole blood was collected in cfDNA blood collection tubes (Streck) and processed within 24 hours via a double spin protocol: first at 1,600 g for 20 minutes to separate

plasma from the buffy coat and then spun at 14,000 g for 10 minutes to remove cellular debris. Buffy coat and plasma were stored at -80C.

DNA extraction

DNA from saliva, buffy coat and whole blood was extracted using the QIAamp DNA Blood mini kit (Qiagen) or the DNEasy Blood and Tissue kit (Qiagen) according to the manufacturer's protocol. cfDNA from plasma was extracted using the QIAamp Circulating Nucleic Acid Kit (Qiagen). DNA was quantified using a Quant-it (Qubit) and Tapestation (Agilent). Input mass was 100 ng of buffy coat or saliva DNA, and for cfDNA the total mass resulting from 3-5 mL plasma was used.

Sequencing

Sequencing libraries were generated using the KAPA HyperPrep kit (Roche) and captured with probes from Integrated DNA Technologies, consisting of the full length of the CH assay genes (*DNMT3A, TET2, ASXL1, TP53, PPM1D, SF3B1, SRSF2, IDH1, IDH2, JAK2, BCOR, MPL, U2AF1, U2AF2, ZRSR2, CBL, CHEK2, GNAS* and *GNB1*), using the SeqCap® EZ Hybridization and Wash Kit (Roche) according to the manufacturer's protocol. xGen Duplex Seq Adapters (Integrated DNA Technologies) containing dual unique molecular indexes (UMIs) were used. Libraries were pooled and sequenced at standard depth (1,000X) on a Miseq (Illumina), and at high depth (10,000X) on a Novaseq (Illumina).

Bioinformatics & error score

FASTQ files were trimmed using Trimmomatic (v0.36) (2) and aligned to GRCh37 using Bowtie2 (v2.3.5) (3). Coverage was calculated using Bedtools (v2.27.1) (4). UMIs were collected and error correction was performed to generate consensus collapsed FASTQs using fgbio (v0.8.0). Error-corrected sequences were realigned using Bowtie2 and variant calling was performed using Vardict (v1.5.8). SNPs and common variants were annotated using SnpEff (v4.3), dbSNP common (v9606_b151), gnomAD (r2.1.1) and clinvar (v20190408). The number of somatic single base substitution (SBS) variant calls <5% VAF with at least 3 variant reads was normalized against the mean distinct coverage to generate a background error score. The default minimum required VAF of 0.2% was adjusted per SBS type as the background error score increases using a log-linear model evaluating the relationship between VAF and count of variants observed per VAF level.

Each variant call was assigned a confidence score using a logistic regression model which estimates likelihood of reproducibility adjusting for variant depth, mutant allele fraction, mutation type, and the background error rate in the sample. This model was trained on an SBS/indel variant set (n=102,163 total; n=262 true positives) labeled for reproducibility based on a replicate analysis, ranging in VAF from 0.09% to 56% (median VAF 0.61%). Utilizing this model in the bioinformatics pipeline, a minimum confidence score of 25 was required for reporting.

Limit of blank

The limit of blank (LoB) calculates the percentage chance of a false positive call in the assay. The LoB was estimated using 70 samples from individuals without cancer, MDS or CH. Mutations likely to be germline events were defined as between 40-55% VAF. LoB was calculated separately for 23 samples run at high depth and 47 at standard depth.

Limit of detection

The limit of detection (LoD) is the lowest frequency at which a variant can be detected consistently (with a 95% detection rate). VAFs from 0.2% - 2.0% were simulated *in silico* with a background score of 0.50 for 1,000X and 3,500X unique distinct coverage (10,000X raw coverage). LoD was estimated per mutation type with 500 simulated variants per parameter configuration. A minimum confidence score of 25 was used to convert positive observations to an overall hit rate. The C95 LoD was derived using a probit model to the hit rate distribution for each SBS type, as well as the non-SBS category of variants.

Two CH buffy coat samples were diluted serially with normal samples to 10% and 1% dilutions and sequenced in triplicate at standard depth (~1000X) and high depth (10,000X, 3,500X collapsed coverage). The number of correctly called mutations was recorded for each percentage to determine the experimental LoD.

Precision

Analysis of the variation of samples within the same run. Five samples were run in multiple replicates on the same sequencing run for a total of 29 replicates. 61 mutations were analyzed in total. For high depth, 3 samples were run twice on the same sequencing run for a total of 6 replicates and 14 mutations were analyzed in total.

Reproducibility

Analysis of the variation of samples across different runs. 17 samples were run across 72 replicates, with a minimum number of 2 replicates per sample and 136 mutations were analyzed in total. For high depth, 3 samples were run across 7 replicates and 16 mutations were analyzed in total.

Analytical accuracy

Analytical accuracy was calculated by comparison to clinically validated assays. Samples with orthogonal MSK-IMPACT or MSK-IMPACT-Heme were compared for mutations and VAFs (1, 5). A maximum time of 12 months was allowed between the CH panel and the orthogonal assay.

Multi-sample integration for variant detection

Statistical modeling of multi-biospecimen variant observations was compared to the performance of a single biospecimen using the previously described logistic regression model with a binomial confidence score model incorporating two or three biospecimens per sample. Multi-biospecimen evaluation of each unique variant observed utilized individual biospecimen estimates of the cumulative density function based on a binomial distribution given: 1. The observed variant reads, 2. The total coverage at that position, and 3. An assumed baseline error rate of 0.2%. The probabilities of observing each specific number of variant reads (or higher) per biospecimen are multiplied to estimate the overall Type I error rate across the sample set. The Type I error rate is then adjusted for multiplicity using a Bonferroni correction and converted to a confidence score 100*(1-overall adjusted Type I error rate) and positive result calling was set at confidence scores >25. Multi-biospecimen simulations for evaluation of sensitivity reflected random variant selection from eight VAF bins (lowest bin from [0.05-0.1%] to highest bin [10-20%]; n=1,000 variants per bin) and simulating 1,000X coverage per variant position across one to three samples.

Multi-sample simulations for evaluation of specificity simulated a panel with 10,000 positions for one to three biospecimens at coverages ranging from 500X to 10,000X, with a baseline error rate of 0.19%. Non-simulated data were based on a total of 63 individuals, 25 of which had three biospecimens and 38 of which had two biospecimens.

Statistics

Sample types (PBMC/buffy coat, saliva, cfDNA) were compared using paired t-tests. SBS types were compared using Mann-Whitney tests. A significance level of $p < 0.05$ was used.

Study Approval

Patients and healthy control individuals were consented under IRB 12-245 or 06-107 and analysis was done under IRB 18-288 and 21-036 at MSKCC, New York, NY, USA. Written informed consent was received prior to participation.

Data availability

The raw sequencing data are protected and are not available due to privacy laws, however we have included a supplementary file of Supporting data values.xls is included with mutation data listed for all samples, under the tab 'Mutation data'. Data for all figures is included in the Supporting data values xls file.

Supplementary References

1. Zehir, A. et al. Mutational landscape of metastatic cancer revealed from prospective clinical sequencing of 10,000 patients. *Nat Med* **23**, 703–713 (2017).

2. Bolger, A. M., Lohse, M. & Usadel, B. Trimmomatic: a flexible trimmer for Illumina sequence data. *Bioinformatics* **30**, 2114–2120 (2014).

3. Langmead, B. & Salzberg, S. L. Fast gapped-read alignment with Bowtie 2. *Nature Methods* **9**(4), 357–359 (2012).

4. Quinlan, A. R. & Hall, I. M. BEDTools: a flexible suite of utilities for comparing genomic features. *Bioinformatics* **26**, 841–842 (2010).

5. Ptashkin, R. N. et al. Prevalence of clonal hematopoiesis mutations in tumor-only clinical genomic profiling of solid tumors. in *JAMA Oncology* **4**, 1589–1593 (American Medical Association, 2018).

Supplementary Figure and Table Legends

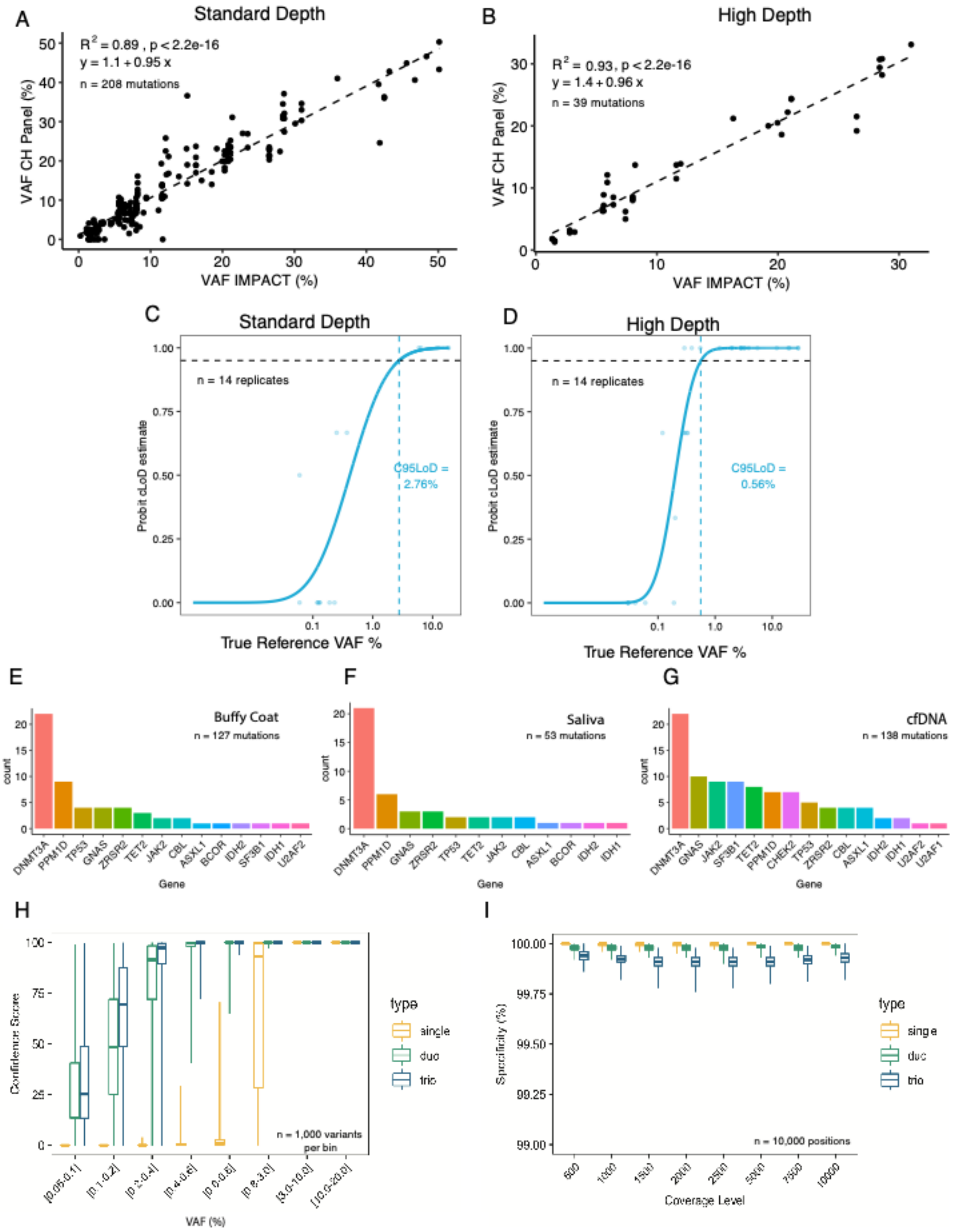
A
Standard Depth
R^2 = 0.89 , p < 2.2e-16
y = 1.1 + 0.95 x
n = 208 mutations
VAF CH Panel (%)
VAF IMPACT (%)
B
High Depth
R^2 = 0.93, p < 2.2e-16
y = 1.4 + 0.96 x
n = 39 mutations
VAF CH Panel (%)
VAF IMPACT (%)
C
Standard Depth
n = 14 replicates
C95LoD = 2.76%
Probit cLoD estimate
True Reference VAF %
D
High Depth
n = 14 replicates
C95LoD = 0.56%
Probit cLoD estimate
True Reference VAF %
E
Buffy Coat
n = 127 mutations
count
DNMT3A PPM1D TP53 GNAS ZRSR2 TET2 JAK2 CBL ASXL1 BCOR IDH2 SF3B1 IDH1 U2AF2
Gene
F
Saliva
n = 53 mutations
count
DNMT3A PPM1D GNAS ZRSR2 TP53 TET2 JAK2 CBL ASXL1 BCOR IDH2 IDH1
Gene
G
cfDNA
n = 138 mutations
count
DNMT3A GNAS JAK2 SF3B1 TET2 PPM1D CHEK2 TP53 ZRSR2 CBL ASXL1 IDH2 IDH1 U2AF2 U2AF1
Gene
H
Confidence Score
type
single
duo
trio
n = 1,000 variants per bin
VAF (%)
I
Specificity (%)
type
single
duo
trio
n = 10,000 positions
Coverage Level

Supplementary Figure 1: CH assay performance for individual samples and samples combined as trios

(**A**) Standard depth and (**B**) high depth scatter plots showing high accuracy between MSK-IMPACT and CH Panel VAFs for buffy coat samples. Probit C95 LOD estimates for (**C**) Standard depth and (**D**) high depth. Dashed line represents calculated LOD with 95% confidence. **(E)** Prevalence of mutations in buffy coat, **(F)** saliva and **(G)** cfDNA. (**H**) Simulated sensitivity when incorporating one (single – yellow), two (duo – green) or three (trio – blue) biospecimens to establish a mutation call. (**I**) Specificity for each model of mutation calling, calling with one biospecimen has limited sensitivity but high specificity whereas increasing the sensitivity by including additional biospecimens slightly decreases specificity. *VAF – variant allele fraction, box – interquartile range, middle bar – median, whiskers – minimum and maximum values, dots – outliers.*

Standard Depth (1000X)	**Total Replicates Analyzed**	**Total Variants Present**	**Variants Correctly Detected (%)**	**Mean Distinct Coverage**
Within Run Precision	29	61	61 (100%)	888X
Between Run Reproducibility	72	136	132 (97.1%)	873X
Accuracy	117	208	197 (94.7%)	899X

High Depth (10,000X)	**Total Replicates Analyzed**	**Total Variants Present**	**Variants Correctly Detected (%)**	**Mean Distinct Collapsed Coverage**
Within Run Precision	6	14	14 (100%)	1982X
Between Run Reproducibility	7	16	16 (100%)	3098X
Accuracy	17	39	39 (100%)	2907X

Supplementary Table 1: CH assay performance statistics

Precision and reproducibility data at standard and high depth.

Type	1000X coverage	10,000X coverage
A>C	0.9	0.3
A>G	1.0	0.3
A>T	0.9	0.3
C>A	1.1	0.4
C>G	1.2	0.5
C>T	0.9	0.3
nonSBS	0.6	0.6

Supplementary Table 2: Limits of detection for the CH assay

Probit fit estimated limit of detection with a 95% confidence interval. Values represent % variant allele fraction.

Sample ID	Bone Marrow Blasts	Peripheral Blasts	Treatment History at Time of Sampling
AML1	83%	30%	7+3 induction
AML2	53%	42%	untreated
AML3	60%	77%	7+3 induction, MEC
AML4	76%	16%	untreated
AML5	77%	8%	Decitabine, BMT
AML6	37%	19%	Decitabine
MDS1	4%	1%	untreated
MDS2	3%	None reported	untreated
MDS3	2%	None reported	Procrit and/or darbepoetin
MDS4	8%	None reported	untreated
MDS5	6%	None reported	untreated

Supplementary Table 3: **AML and MDS patient clinical data**

Clinical data for AML and MDS patients *(BMT = bone marrow transplant, MEC = Mitoxantrone, Etoposide, and Cytarabine).*

Funding and Acknowledgments

Funding was provided by Swim Across America and NIH P30 CA008748. We would like to acknowledge the Clinical Bioinformatics Group at MSKCC, New York, NY, USA, with their assistance in obtaining access to the healthy donor cfDNA samples used in this manuscript.

Author contributions

Conceptualization: CMS, SP, JRW, JH, LAD

Methodology: CMS, SP, JRW, LAD

Investigation: CMS, SP, JRW, MP, EG, DG, RB, KH, OA, MBF

Resources: KB, RL

Validation: CMS, SP, JRW

Formal analysis: CMS, SP, JRW

Visualization: CMS, JRW, OA

Funding acquisition: LAD

Project administration: CMS, MLE, MP, KH

Supervision: LAD

Writing – original draft: CMS, JRW

Writing – review & editing: CMS, SP, JRW, LAD
